# Community perceptions, acceptability, and the durability of house screening interventions against exposure to malaria vectors in Nyimba district, Zambia

**DOI:** 10.1186/s12889-024-17750-4

**Published:** 2024-01-24

**Authors:** Kochelani Saili, Christiaan de Jager, Freddie Masaninga, Brian Chisanga, Andy Sinyolo, Japhet Chiwaula, Jacob Chirwa, Busiku Hamainza, Emmanuel Chanda, Nathan N. Bakyaita, Clifford Maina Mutero

**Affiliations:** 1 International Centre of Insect Physiology and Ecology (icipe), Nairobi, P.O. Box 30772-00100, Kenya; 2 School of Health Systems & Public Health, University of Pretoria Institute for Sustainable Malaria Control, University of Pretoria, Pretoria, South Africa; 3 World Health Organization, Lusaka, Zambia; 4 Development Economics Group, Wageningen University and Research, Wageningen, Netherlands; 5 National Malaria Elimination Centre, Lusaka, Zambia; 6 World Health Organization, Regional Office, Brazzaville, Congo

**Keywords:** Community perceptions, Acceptability, Durability, House screening, Malaria, Mosquitoes, Zambia

## Abstract

**Background:**

House screening remains conspicuously absent in national malaria programs despite its recognition by the World Health Organization as a supplementary malaria vector-control intervention. This may be attributed, in part, to the knowledge gap in screen durability or longevity in local climatic conditions and community acceptance under specific cultural practices and socio-economic contexts. The objectives of this study were to assess the durability of window and door wire mesh screens a year after full house screening and to assess the acceptability of the house screening intervention to the participants involved.

**Methods:**

This study was conducted in Nyimba district, Zambia and used both quantitative and qualitative methods of data collection and analysis. Both direct observation and questionnaires were employed to assess the durability of the screens and the main reasons for damage. Findings on damage were summarized as percentages. Focus group discussions were used to assess people’s knowledge, perceptions, and acceptability of the closing eaves and house screening intervention. Deductive coding and inductive coding were used to analyse the qualitative data.

**Results:**

A total of 321 out of 400 (80.3%) household owners of screened houses were interviewed. Many window screens (90.3%) were intact. In sharp contrast, most door screens were torn (*n* = 150; 46.7%) or entirely removed (*n* = 55; 17.1%). Most doors (*n* = 114; 76%) had their wire mesh damaged or removed on the bottom half. Goats (25.4%), rust (17.6%) and children (17.1%) were cited most as the cause of damage to door screens. The focus group discussion elicited positive experiences from the participants following the closing of eaves and screening of their windows and doors, ranging from sleeping peacefully due to reduced mosquito biting and/or nuisance and having fewer insects in the house. Participants linked house screening to reduced malaria in their households and community.

**Conclusion:**

This study demonstrated that in rural south-east Zambia, closing eaves and screening windows and doors was widely accepted. Participants perceived that house screening reduced human-vector contact, reduced the malaria burden and nuisance biting from other potentially disease carrying insects. However, screened doors are prone to damage, mainly by children, domestic animals, rust, and termites.

**Supplementary Information:**

The online version contains supplementary material available at 10.1186/s12889-024-17750-4.

## Background

Malaria is endemic throughout Zambia and is a major public health concern [1, 2]. To reduce the malaria burden, Zambia’s National Malaria Elimination Program (NMEP) has developed a multi-pronged approach of combined vector-control interventions, mainly long-lasting insecticidal nets (LLINs) and indoor residual spraying [IRS], prompt malaria diagnosis using rapid diagnostic tests (RDTs), treatment using artemisinin-based combination therapies (ACTs) and strengthening information systems for quality and timely reporting of infections [3–6]. As a result of these interventions, the national malaria prevalence measured in children under the age of five decreased to as low as 9% by 2018 [7]. However, by 2021, the national parasite prevalence rate was reported to be 29% for children younger than five years [8].

The increased prevalence observed in the 2021 nationwide malaria indicator survey (MIS), most probably highlights the negative impact of the COVID-19 pandemic on malaria service delivery during the years 2019–2021 [9, 10]. Further, it underscores the increased need for additional innovative tools in malaria vector-control in order to achieve elimination of the disease [11]. In this connection, the WHO-recommended insecticide-based vector-control interventions used in Zambia, IRS and LLINs, are faced with serious challenges particularly, the development of insecticide resistance among the local vector populations [12–17]. Thus, insecticide resistance may undermine the continued efficacy of IRS and LLIN use against malaria vectors [18], a situation which calls for urgent introduction of new supplementary vector-control tools [11, 19].

In spite of having been recommended by WHO as a supplementary vector-control intervention [20], house screening remains conspicuously absent in the Zambia national malaria program [21, 22]. This is despite evidence showing that in rural Zambia, human-vector contact occurs primarily indoors [23] and Zambia’s reported past success of malaria control with house screening as a supplementary method [24, 25]. The current omission of the intervention in the national program may be attributed to the limited evidence available on the additional benefits of house screening when used in combination with LLINs in different local malaria transmission settings [21, 22, 26]. Furthermore, knowledge on the durability or longevity of house screens when used under local climatic conditions is also limited. There is also a paucity of data on community acceptance under specific cultural practices [20].

This study was part of a larger randomized controlled study evaluating the effectiveness and impact of community-based house screening as a complementary malaria vector-control tool, conducted in rural south-east Zambia [27, 28]. As part of that trial, the intervention group consisted of 400 households provided with LLINs and fine wire mesh screens to stop mosquito entry. Eaves and smaller holes were closed with locally made bricks and mud used for house construction [28]. Wooden frames were fitted with wire mesh in front of the main door externally using hinges, while the edges of these frames were fitted onto the wall by a mixture of mud and cement. These wire gauze/mesh on the houses permitted ventilation. Community health volunteers were used to sensitize the community while the artisans (carpenters and bricklayers) were hired locally from within the study community to increase community acceptability [28].

The objectives of this study were to assess the durability of the window and door screens a year after screening; assess peoples’ perception towards malaria and prevention methods and to assess the acceptability of the of house screening intervention by the participants involved.

## Methods

### Study area

**Fig. 1:**
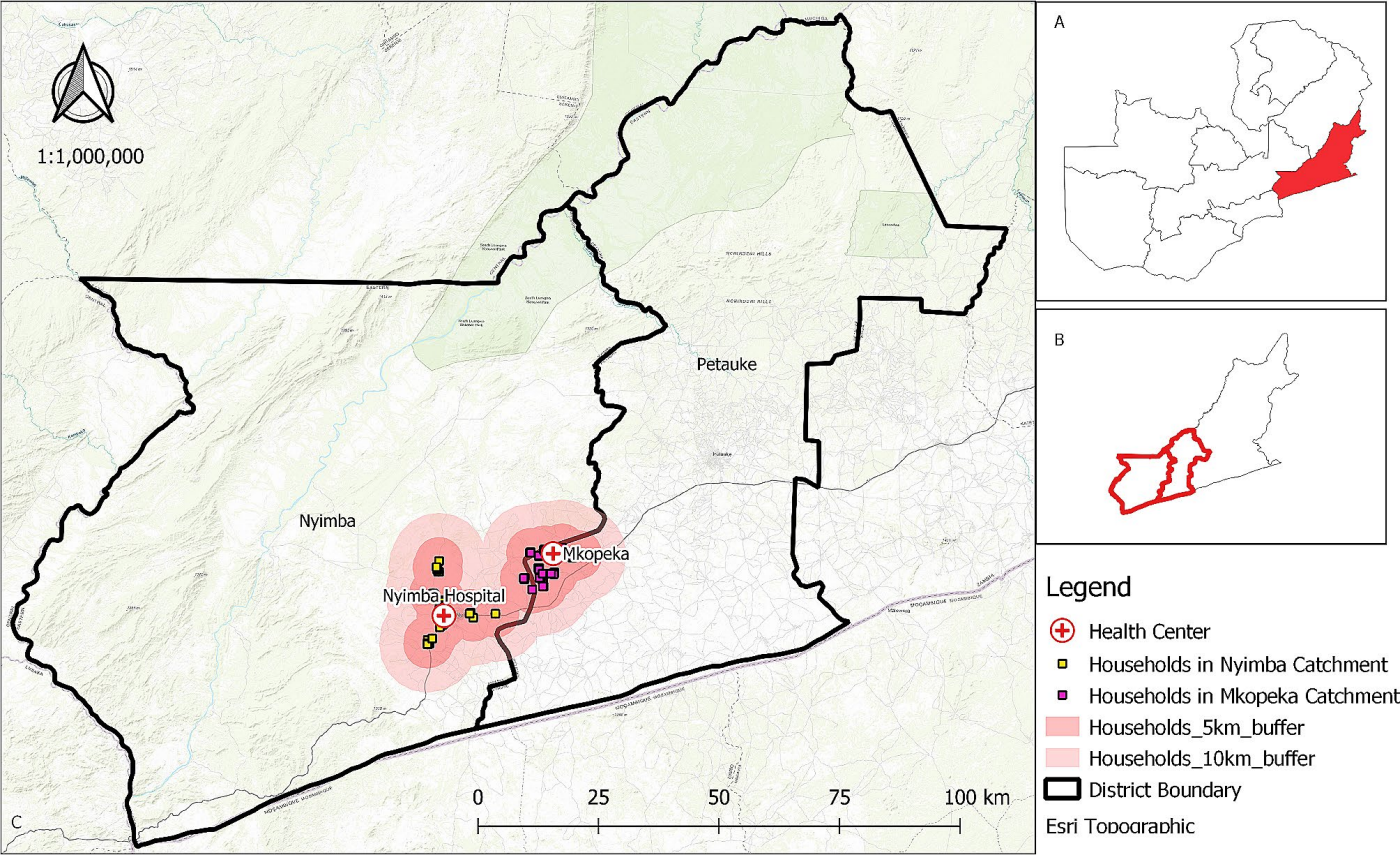
Nyimba district showing the location of households that participated in the house screening. Insert: Map of Zambia showing the location of Nyimba district

The study was conducted in Nyimba district, located in the Eastern province of Zambia (4° 21′ 0″ S; 30° 35′ 0″ E) in December 2020 and January 2021 (Fig. 1). The study area has been described in detail elsewhere [27, 29]. Malaria in this area is endemic and transmission is perennial although it is highest after the end of the rain season, between March and May [7]. Malaria cases are almost entirely attributable to *Plasmodium falciparum* [7]. The major economic activity in the area is subsistence agriculture. Maize and groundnuts are the major crops grown. Other crops cultivated include sunflower, soya beans and cotton. Cattle and goats are kept as part of animal husbandry [30].

### Study design

The study used a mixed qualitative and quantitative method study design. It initially involved direct observation, followed by a questionnaire to assess the durability of the wire mesh screens and main reasons for wear and tear. To enhance our understanding of the social and cultural phenomenon for the damages and/or removal, focus group discussions (FDGs) were held. FDGs were opted for because of the depth they guarantee in understanding a social phenomenon. The FGDs were used to first, assess people’s knowledge, attitude, and perception of the local malaria situation. Second, to assess the knowledge, perceptions, and acceptability of the house screening intervention. This was important as some householders refused to respond to the question about the damage to the door screens resulting in invalid responses or missing values.

### Sample size

The sample size used in this study has been described elsewhere [27]. Briefly, the sample size was derived from simulation models described in Hayes and Bennet et al. [31] for incidence rates and routine data collected from all health facilities in Nyimba district in Zambia at an estimated incidence rate of 0.312 cases per person from January to June 2019. It was estimated that to detect a reduction of 35% on malaria incidence, with 80% power at the 5% significance level, 338 houses were required per study arm [32]. A total of 400 households with one child each were recruited per treatment arm with additional households enrolled to account for households lost to follow-up.

### Durability surveys

A questionnaire was used to assess the condition of the installed wire gauze on both the windows and the doors (see Additional file 1). Data was collected from 321 out of the 400 (80%) participating households, thus measuring the larger proportion of the intervention population. The questionnaire was pre-tested on 20% (*n* = 80) of the screened households from the two study sites. During the pilot study, it was determined that at least one year after the installation, the wooden framework of doors and windows, the mortar holding the doors and window frames in place and the mortar that filled the eaves were still intact. This was thus, not included in the data collection tool.

**Fig. 2:**
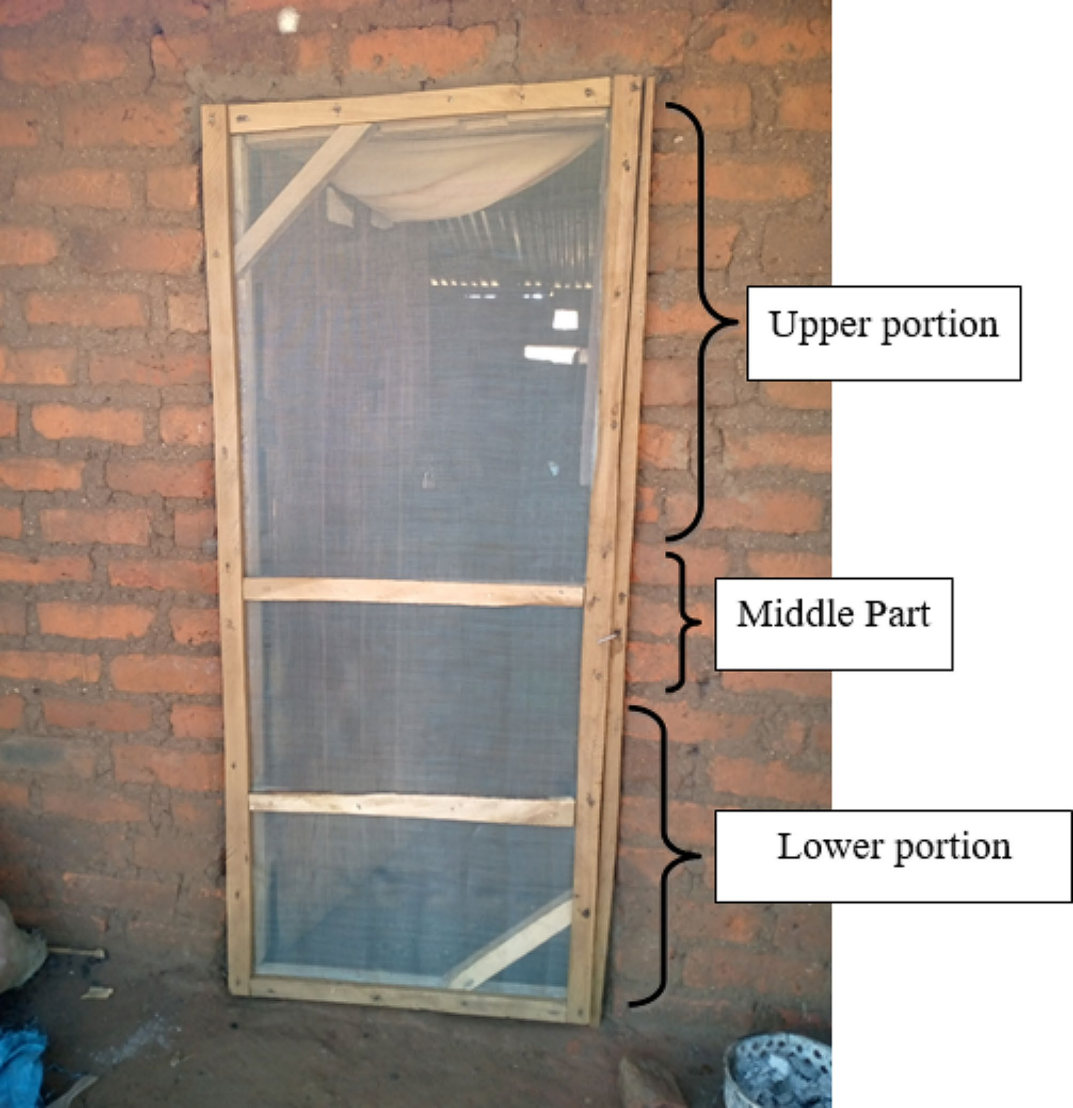
A newly installed door screen showing the three portions considered in the questionnaire to assesss damage

To assess the condition of the doors, three broad categories were used; “intact”, “torn”, “removed”. The screened door was considered “intact” when the wire gauze did not have any visible damage or holes or tear larger than 2 cm in diameter. The screen door was considered “torn” if the wire gauze was detached from the wooden plank or had a hole/s larger than 2 cm in diameter. If the wire gauze was removed or torn, the householder was interviewed to understand the reasons of the removal or tearing. For doors, an additional section was added to understand which part of the door was affected the most: “bottom”, “middle”, “top” or “entirely removed”. This is illustrated in Fig. 2.

### Focus group discussion

Focus group discussions were conducted to assess participants’ knowledge, perceptions, and acceptability of closing of eaves and screening windows and doors as a malaria vector-control intervention. The interviews were conducted by the research team. Before the interviews, all data collectors received a one-day training. Training included an overview of the study, review of the interview guide (Additional file 2), with an emphasis on the main objective of the focus group discussions, and qualitative interviewing techniques.

Fourteen focus group discussions were held. This corresponded to 14 out of the 20 villages that had both the house screening intervention implementation and entomological surveillance [27]. In each village, six household heads (or their proxies) that had consented to their house being screened and six that had either not given consent or missed out entirely due to ineligibility or absenteeism during the screening period, were interviewed. Community health workers (CHWs) supported the selection of households. Before the interviews, all study participants were notified of the date, place, and time of the meeting for holding these FGDs. The FGDs were conducted at community centric places like schools, churches, or health facilities. All participants were 18 years and above and composed of both sexes. FGDs took between 1 and 2 h per session. All interviews were conducted in Nsenga, the most widely spoken language in the area. All data collection took place in December 2020.

During the FGDs, we used participatory rural appraisal (PRA) approaches to determine the community’s perception of the malaria situation, display of symptoms among children, and confirmed malaria by RDT. Using 10 stones to represent children, we asked at least three participants to separately put stones in boxes labeled “malaria positive” and “malaria negative”, as confirmed by RDT. These stones would be proportionate to the individual’s perception of the number of children either malaria-positive or negative. We then asked all participants to confirm which was most accurate. This was repeated for children “displaying malaria symptoms only”.

### Data analysis

This was a descriptive survey. All data were entered and stored into an Excel spreadsheet (Microsoft Office 2018). Findings on damage were summarized as percentages and proportional differences in the damages on the doors and windows determined by Pearson’s chi- square (χ^2)^ at 0.05 significance.

At the end of each day of interviews during the data collection period, notes were taken, and discussions were held with the entire research team members as part of the preliminary data analysis. All data from the focus group discussions were audio-recorded, transcribed verbatim, and translated into English by a research assistant.

Thematic analysis was used to analyse the data. The transcripts were coded one of the authors and shared for comments and agreement on a common coding framework to the other authors. Both deductive coding and inductive coding were used. The deductive codes were derived from pre-established codes and were based on the interview guide (Additional File 2). Inductive coding was based on codes that emerged during the analysis process and were derived from the participants own words [33]. Key themes in the coding framework included the community’s knowledge and perception of malaria prevalence and symptoms in children; malaria preventive methods; knowledge, perceptions, and experiences with house screening, barriers and facilitators of house screening and sustainability of the house screening intervention. These themes were framed around the Health Belief Model (HBM), a framework commonly used to explore compliance to health interventions. It can be used to interpret perceptions, acceptance, and usage of a health intervention [34, 35]. The model has six elements to explain and predict preventive health behaviours: (1) perceived susceptibility of the individual to the condition (2) perceived severity of the condition, (3) perceived benefits, (4) perceived barriers, (5) self-efficacy which is the conviction that one can successfully execute the health behaviour and (6) cues to action which trigger the readiness [34, 36]. The themes in this study were derived from these elements. This is explained in Table 1.

**Table 1:** Main themes from the qualitative study

Theme	Data supporting the theme/ sub-themes	Researchers’ interpretative summary
Knowledge, perceived susceptibility, and severity of malaria in children	Basic knowledge of malaria	• Community members theoretical understanding of the cause of malaria • Perception of malaria prevalence in comparison to previous years and • Linkage between theoretical understanding and perceived reasons for increase or decrease of malaria in children
	Knowledge of symptoms of malaria	
	Perception of the prevalence or how common malaria symptoms were and reasons for increase or decrease	
Malaria prevention methods	Identification of core vector-control methods i.e., LLINs and IRS	Basic knowledge relating to malaria vector-control interventions
	Identification of personal protection measures	
Knowledge and perceived benefits of house screening	What house screening entails	• Community members theoretical and practical understanding of house screening as a supplementary intervention • Positive experiences
	General perceptions, experiences, and concerns	
	Complementary role house screening plays in malaria prevention	
Barriers of house screening	Lack of ventilation, heat, poor lighting, termites and/or rust on screened houses	• Motivating and demotivating factors to community involvement • Negative experiences
**Self-efficacy**	The appropriateness of house screening as a supplementary intervention	Community member approval or disapproval of house screening
Cues to action	Considerations and challenges	The willingness to implement house screening
	Community ownership	The willingness to maintain or repair damaged screens

## Results

### Condition of window screens

Overall 321 (80.3%) of the 400 houses that were screened were observed and household owners interviewed. Table 2 summarizes the findings of the condition of the window screens. There was significantly higher proportion of intact window screens than damaged (torn or removed) (χ^2^ = 490, df = 1, *P* < 0.01 ) at the time of the survey. Reasons given for the torn window screens included poor workmanship, rust and children poking the screens with sticks and/or wires.

**Table 2:** Condition of the wire mesh used in screening the windows

Condition of screened window	Frequency	Percent
Removed	1	0.3%
Torn	14	4.4%
Intact	289	90.3%
Invalid/missing values	17	5.0%
**Total**	**321**	

### Condition of the door screens

**Fig. 3:**
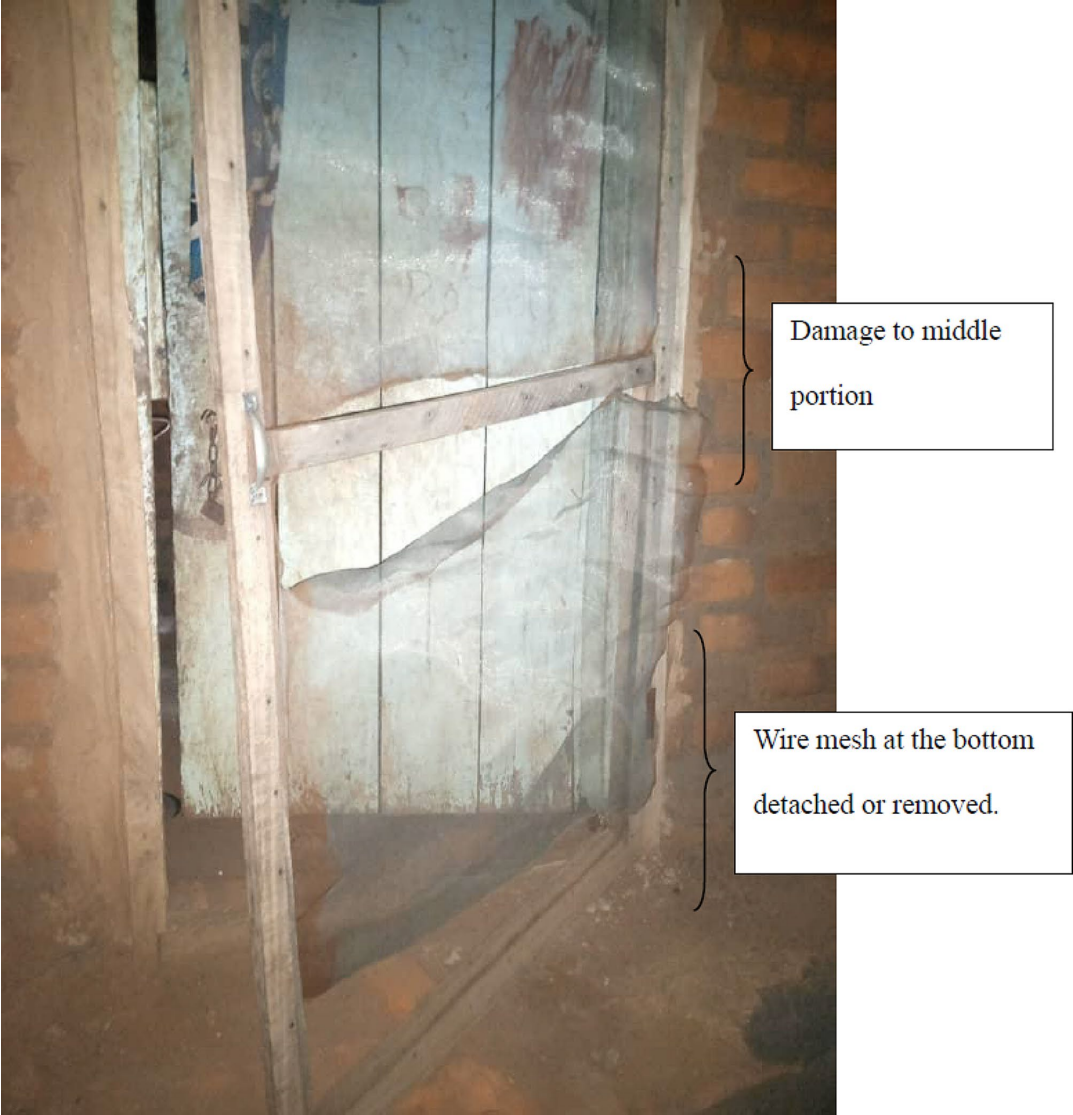
Damaged door screen showing the portions that were damaged the most

**Fig. 4:**
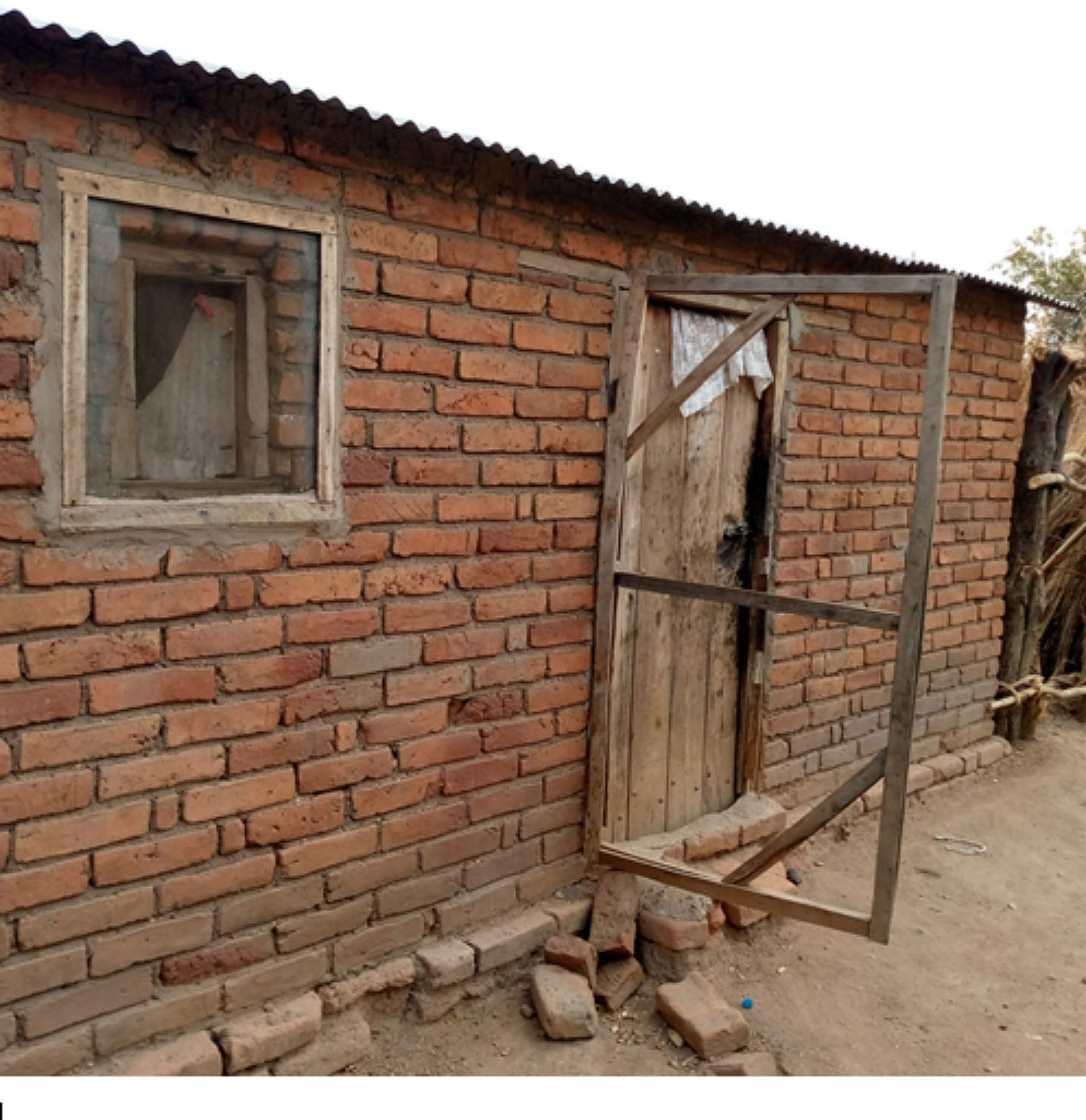
Completely removed wire gauze on a door screen frame

The wooden framework and the mortar holding the doors was in place for most doors. However, we found that most of the screens were either torn (*n* = 150; 46.7%) or removed (*n* = 55; 17.1%) (Table 3). There was significantly higher proportion of damaged wire mesh on door screens (torn or entired removed) than intact ones (χ^2^ = 52.1, df = 1, *P* < 0.01 ) at the time of the survey. For most doors (*n* = 114; 76%), the bottom half was torn or removed. This is summarized in Table 4 and illustrated in Figs. 3 and 4.

**Table 3:** Condition of the wire mesh used in screening the doors

Condition of door screen	Frequency	Percent
Entirely removed	55	17.1%
Torn	150	46.7%
Intact	113	35.2%
Invalid/ Missing values	3	0.9%
Total	**321**	

**Table 4:** Damage to screened doors

Portion of the door screen	Frequency	Percent
Bottom portion	114	76.0%
Middle part	25	16.7%
Upper portion	11	7.3%
**Total**	**150**	

Goats were identified most frequently (25.4%) as the cause of damage, more specifically, to the bottom half of the door screens. According to most household heads, this happened when goats attempted to enter the house to eat stored food. Rust and children *“running in and out of the house”* were the second and third most frequently cited causes of damage respectively. Destruction of the wood by termites and poor workmanship was also cited by the households as another cause of door screen damage. However, some householders refused to respond to the question about the damage to the door screens resulting in invalid responses or missing values (Table 5). This in part prompted the focus group discussions.

**Table 5:** Cited reasons for damage or removal of door screens

Reasons for damage	Frequency	Percent (%)
Goats	52	25.4%
Rust	36	17.6%
Children	35	17.1%
Poor workmanship	23	11.2%
Termites	4	2.0%
Cattle	2	1.0%
Other	13	6.3%
Invalid/missing values	40	19.5%
Total	205	100

### Focus group discussion

In total there were 162 participants spread across 14 meetings. On average, each meeting had 11 attendees. A total of 80 females and 82 males attended. Of these, 91 had houses that were not screened (control) and 71 had screened houses (intervention). The average age of the participants was 39 years. Other demographics of the participants are shown in Table 6.

**Table 6:** Demographics of study participants

Characteristic	Nyimba Urban	Mkopeka	Total (%)
**Gender**			
Male	43	39	82 (50.6)
Female	37	43	80 (49.4)
**Age**			
Average	38.1	39.9	
18–24	9	6	15 (9.3)
25–44	49	45	94 (58.0)
≥ 45	22	31	53 (32.7)
**Education**			
Informal	17	18	35 (21.6)
Primary	48	44	92 (56.8)
Secondary	15	16	31 (19.1)
Tertiary	3	1	4 (2.5)

### Knowledge, perceived susceptibility, and severity of malaria in children

Symptoms of malaria were readily identifiable by the participants in all the 14 focus group discussions. Participants identified fever, directly translated as “*body hotness”* in the local language, as a key malaria symptom. Vomiting, chills, shivering, loss of appetite, lethargy and fatigue, blood shot eyes or *“red eyes”, “pain in the body joints”* were mentioned as some common symptoms. Convulsions were also readily identified as a symptom of severe malaria due to delayed treatment.

*“Sometimes, you cannot see any of those symptoms these ladies have mentioned. But you see your child not playing with his friends, not active.. when taken to the clinic you find that they have malaria”*- male respondent, Nyakozolo village.

*“Sometimes a child [gets convulsions] when you delay taking them to the clinic”*, male respondent, Chambula village.

**Fig. 5:**
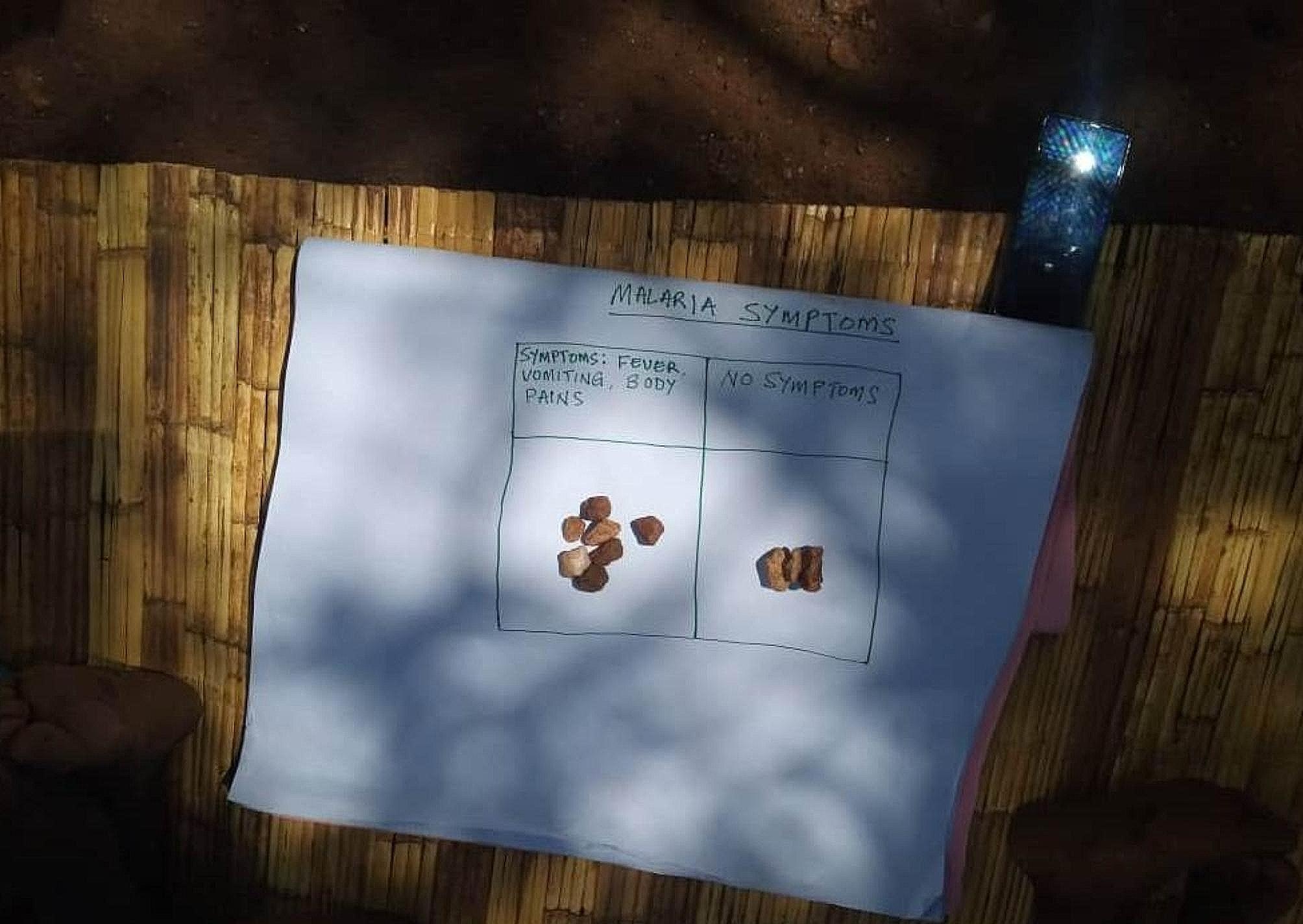
Identifying proportions of children displaying malaria symptoms using PRA methods

Once identified, we used participatory rural appraisal (PRA) methods to determine the community’s perception of the malaria symptoms and confirmed malaria in children. Using 10 stones to represent children, we asked at least three participants to proportionate the stones according to children displaying malaria symptoms. This is illustrated in Fig. 5. We then asked all participants to confirm the most accurate.

Most of the community members revealed that children showed malaria symptoms but tested negative when tested for malaria. A further probe for proportions of confirmed malaria using PRA methods had most participants placing more stones in the malaria “negative box”. The ratio of positive to negative confirmed malaria as represented by the stones was generally agreed at 3 to 7.

*“.. sometimes, my child would have fever but when taken to the clinic, they would not find malaria. That leaves me wondering what caused the fever in the first place.”-*female respondent, Chambula village.

*“These days when you take five children to the clinic, you would find only one has malaria.“-* female respondent, Sikatoba village.

Participants felt that malaria cases in the community had reduced in comparison to the previous years. This was attributed to the distribution of LLINs, house screening or “*mosquito screens”*, IRS and health education given to the community member through the health facilities.

*“The other thing that has led to the reduction in the number of [malaria] cases is the introduction of mosquito screens. Once the mosquito screens are installed mosquitos do not enter the house.“-* male respondent, Kalunga village.

### Malaria preventive methods

In most FGDs community members identified at least three malaria preventive methods; LLINs, IRS, and house screening. Burning a special type of grass/herb, traditionally known as *“mutanda imbu”*, which directly translates *‘chase the mosquitoes’* was frequently mentioned. Some participants however mentioned they no longer use it.

*“To be honest we no longer use that mutanda imbu.. not anymore. Maybe in the olden days. Now we just sleep under mosquito nets”-*male respondent, Nyakazolo village.

Many participants also mentioned *“mosquito coils”* and *“body creams to keep mosquitoes away”* i.e., spatial and body repellents respectively. Included were some personal protective measures that reduced mosquito bites such as sitting near a smoking fire or wearing long sleeved shirts and long trousers. Environmental manipulation such as getting rid of stagnant water and keeping grass short were frequently mentioned.

*“Burying all ditches holding still water in the yard because that is where mosquitos mostly breed from”-*male participant, Mkopeka village.

*“We also encourage children to wear long sleeved clothes in the evenings to avoid being bitten. Also, once they give us mosquito nets, we make sure children are nicely tucked in when they go to bed”-* male participant, Lupala village.

### Knowledge and perceived benefits of house screening

Throughout our discussions, participants mentioned hearing about house screening largely through the CHWs who participated in the enumeration (prior to the installation) and during installation. The participants generally referred to the wire mesh as *“ma seifa”*, a local name for the wire mesh used. Many community members acknowledged not to have heard about the house screening intervention or use of the wire mesh on windows and doors for the prevention of mosquito entry before this study. Almost all participants indicated that screening windows and doors prevents malaria by reducing mosquito entry.

*“From my understanding, a mosquito has wings. The holes on the screen are so small such that even if the mosquito manages to put its head through, the wings won’t be able to enter.“-* Male participant, Nyakozola village.

*“Mosquito screens have been helpful, you will find absolutely no mosquitos in the house as long you always close your [door] screens as required.“-* Female participant, Malipa village.

Participants shared their positive experiences after the closing of eaves and screening their windows and doors. These ranged from sleeping peacefully due to reduced mosquito biting and/or nuisance and having fewer insects in the house. Some community members explained the intricate link between house screening, nuisance insects and potential infectious biting from other insects other than mosquitoes such as fleas.

*“We now sleep like kings, peacefully. No slapping mosquitos when we are sleeping. As long as we close the screened doors nicely. It is very helpful.“-* male participant, Ziko village.

*“Screens do not kill rats. Sometimes the rats come with fleas which do not leave the house when the rats go out. The fleas continue biting humans when rats are gone. But with the screens and closed eaves, even the rats do not enter. We want these screens, please”-* female participant, Malipa village.

*“Cockroaches have reduced. During this rainy season, the number of insects coming into the house [being attracted by the light] has significantly reduced.”-* female participant, Malipa village.

Participants linked installing gauze wire during house screening to reduced malaria infection rates in their households and community.

*“I have a child who is 6 years old. Before putting the screens, I was taking him to the clinic every month, sometimes twice a month. But this time he never gets malaria ever since the screens were put. I am very thankful”-* female participant, Sikatoba village.

*“They put my screens last year and after some 2 to 3 months my child stopped getting sick. Even up to now!“*- Female participant, Sikatoba.

*“We used to go to the hospital very frequently. Now, with the screens, we don’t get sick. Before the screens, each one in the family would have malaria. I tell you, malaria would make it’s rounds on us. Now, none of us get malaria”-* female participant, Mtausi village.

*“This past year, the children used to sleep in a house without screens. They would frequently suffer bouts of malaria. But now my children sleep in a house with screens. They never get sick”*-female participant, Chambula village.

Other positive experiences related to the aesthetics i.e., the *“houses with screens looked good”.* Many praised the increased ventilation and lighting resulting from the screening. Overall, lighting and ventilation were not mentioned as a hinderance.

*“We admire how the houses which have mosquito screens look, the windows look fancy”*-female participant with a house without screens, Kapakasa village.

*“Before we used to block the window, with clothes and sacks. Now we allowed those installing the screens to remove some blocks and make the airspace bigger. We have fresh air all the time”-*female participant, Mulira village.

### Self-efficacy

With this background, self-efficacy, the perception, or confidence of respondents towards house screening as an added intervention was measured. Respondents were asked to list in order of effectiveness house screening as a malaria intervention, against ITNs, IRS, spatial repellents, and body repellents (whichever the participants had mentioned earlier). In many cases, house screening as an intervention was second or third choice with ITNs and IRS being preferred or considered more effective. When screening was picked as the second preference, ITNs were always first choice.

We asked the participants to grade the house screening intervention on a scale of 1 to 10, with 10 being the best and one least. In many cases, the house screening received a grading of between 8 and 9 out of 10.

*“I will give the screens 8 out of 10. They are helpful. But I have removed the 20% because they rust easily”*- male participant, Mkopeka village.

*“I will give the screens 8 out of 10; yes 80%! Us as parents, we go out for work or at the farms. The children destroy [the screens], especially the screened doors. The 10% I have removed is because of that and rust.. we end up having big holes. ”*- female participant, Mkopeka village.

### Barriers to house screening

In many focus group discussions, damage, largely due to rusting was perceived as the biggest barrier to the acceptance of house screening. Similar to the durability survey, poor workmanship, goats, and children were mentioned as the top causes of damage particularly to the door screens which would then become unsightly.

*“In my observation, the screens were not properly made. Where the screen is attached to the plank, they made it so tight that if anything bumps into it, the wires dislocate and make a hole. That is why mine is badly damaged.“-*male participant, Lupala village.

*“The people that put the door screens were in a hurry such that they did not do a good job. The door screen fell off within the first month that they installed it.“-* male participant, Ziko village.

*“.. when it rains, water would splash on the screen. After the rust developed, some goats had entered the house and when the children were chasing them, the goats ran into the screen, and it got badly damaged. It does not look nice anymore”-*female participant, Ziko village.

*“In my case, termites damaged the planks holding the screen until the screen was left unsupported.“-* Male participant, Chambula village.

Light, ventilation or heat were not mentioned as inhibitors to the acceptance of the house screening even after thorough probing. Use of the local community health workers for community engagement and local artisans and bricklayers helped with the acceptability of the intervention overall.

### Cues to action

In this study cues to action refers to the participant’s readiness to initiate or maintain house screening. This was measured through a willingness of participants to install and/or maintain the screens in the absence of support from the Ministry of Health or its partners. Recognizing the benefits, house screening as an intervention was well received and recommended with many participants expressing the willingness to buy the material on their own. This was after realising that materials were readily available and commonly used to make locally made sieves used for mealie meal and groundnuts. However, many participants expressed hesitation to install and maintain the screens on their own. This was largely based on their experience with the wire mesh which once rusted, could barely be repaired.

*“How can we even fix them? These are just like the household sieves we use to sieve mealie meal. It’s not possible to only repair a part of it. The only way is to remove it completely and then put another one”- m*ale participant, Mkopeka village.

“*He got wires and hooked them back in place. Later, it was dislocated where the screen touches the plank. After that when you try to repair it, the wires don’t hold because they are rusty.”*- female participant, Kalunga, describing how her husband tried to fix the screening.

In all the focus group discussions, communities requested that there should be clarity who should be maintaining the screens, i.e., either themselves, the Ministry of Health and/or project partners. There was a clear gap in the sense of ownership.

*“If the government, I am talking about the Ministry of Health and partners, makes it clear that these things are [ours] and that [we] should be maintaining them, then we will repair them”-*Lupala village, male respondent.

## Discussion

This study assessed the durability and community knowledge, perception and acceptability of the house screening intervention one year after installation. Our findings reveal that most window screens (90%) were intact or undamaged. However, 17.1% of screened doors had wire mesh entirely removed whilst about half (46.7%) had torn wire mesh. Only 35.2% were intact and fully functional. Studies in Ethiopia [37] and The Gambia [38–40] similarly reported more damage to doors than to window screens. Damage to the doors was mostly caused by domestic animals, (specifically goats), children, rust and termites, similar to the findings of Getewan et al. [37] and Kirby et al. [38]. The highest damage on the screened doors was at the bottom and middle parts as earlier defined. This created two critical barriers to acceptability of the house screening intervention. First was the negative experience resulting from the damage to the screened doors [41]. The focus group discussion echoed information recorded in the questionnaire, namely, that domestic animals, rust and childen were the biggest cause of damage to the wire mesh on the screened doors. Once rusted, the screened doors became unsightly hence householders could remove them completely. A second barrier to acceptability was the inability to repair broken screens. This may in turn affect long term sustainability of house screening by the householders [21]. The inability to repair was due to the rusting of the metallic or wire mesh. Once rusted, this material was practically irreparable.

The above findings revealed impediments to the acceptance of house screening as a supplementary vector-contol intervention. From the results of this study, we therefore suggest the following improvements on the design of the screened doors to prevent entry of mosquitoes inside the house in the rural areas of Zambia. First to replace the wire mesh with polyvinyl chloride (PVC) fibre glass which may be readily available locally. This may increase durability and in the long run reduce the costs associated with damaged screens [21, 42]. Percieved high costs and inability to repair, thus, low sustainability, ranked highly among concerns associated with housing improvement as a supplementary malaria vector-control intervention [21, 26, 43]. The replacement of wire mesh with PVC fibre glass may provide a solution to this. Second recommendation is a hard material for the bottom-half of the door, peharps made of locally available plywood or hardwood. The bottom part of the door was more likely to be damaged from domestic animals and small children running inside and outside of the house. Third, the upper part of the door should be reinforced with larger sized wire (chicken wire) or plank. (see Additional file 3). And fourth, it is recommended that all wood to be treated with anti-termite. Whilst initial costs may be higher, these changes may reduce damages and the need for replacement. This may prove more cost-effective in the long run. The prototype described in The Gambia study [44] could provide further alternatives to the above modifications.

The FGDs revealed universal knowledge of house screening. This could be attributed in part to working closely with CHWs, masons (brick layers) and carpenters from the participating villages within the study area. Involving a local community member in delivering malaria interventions breaks the power differences that may exist between the researchers and the community [45]. This built trust and thus, increased awareness and promoted acceptance [41, 45–47]. House screening was associated with reduced mosquito densities and as a consquence, reduced biting and malaria infections. These findings corroborate with the findings of a parrallel study by Chisanga et al. [47] who showed that house screening significantly reduced self-reported malaria in the study area. Individuals in screened houses reported over 40% less self-reported malaria, 25% less number of sick days and 17.5% episodes of suspected malaria [47].

Further, house screening was readily associated with reduced nuisance from other pests. Participants told of how screening reduced entry of rats, cockroaches, snakes and other insects particularly during the rainy season. Our findings are consistent with those from The Gambia [38] and Malawi [41]. One participant intricately highlighted the added health benefits of house screening with reduced exposure to plague, a flea-borne rodent-associated disease. Nyimba recorded fatal cases of plague in 2015 [48, 49]. This underpins the added benefit of improved housing as a developmental intervention in further reducing the burden of other arthropod-borne diseases such as diarrhoea, plague, lyphatic filariaisis and *Aedes*-transmitted diseases [22, 50, 51]. Other participants, felt their houses *“looked beautiful”* with the screens. These experiences are similar to those described in The Gambia [38] and may highlight yet another motivation to having houses screened with wire mesh.

Another key finding of this study was that house screening was indeed viewed as a supplementary method of preventing malaria by the participants. Community members always ranked ITNs and at times, IRS to be better than house screening. This may imply that house screening would not interfere with use of ITNs and IRS. This is an important finding. The WHO recommends universal access to vector-control, either ITNs or IRS at optimal coverage levels for all populations at risk of malaria in most epidemiological and ecological settings [20]. House screening as an intervention remains supplementary and should not be viewed as a replacement for the core malaria interventions [20].

Light and ventilation were not mentioned as barriers to acceptance. This is similar to findings by Getawen et al. [37] who showed that screening doors and windows did not interfere with either air flow nor lighting. Our findings however, contrast observations from The Gambia [38, 39] and Malawi [41] where some participants complained about poor lighting as a result of the closed eaves and screened doors and windows. Choice, type and design of the mesh on the screens must take into considertion the householders thermal comfort, ventilation and airflow [41, 44, 51, 52]. In this study, householders at times requested typically small air spaces to be increased by the removal of a layer of bricks or the clothes and sacs used to block theses spaces. This allowed more light and greater airflow. This added step by the householder may have further resulted in the co-benefit of reduced acute respiratory diseases [22] and increased acceptabilitity [46]. With increased air flow, adequate lighting and the absence of mosquitoes or other disturbing insects, indeed many could *“sleep like kings, peacefully”.* This information could be included in community engagement key messaging about house screening to increase acceptability [41, 46].

This study had limitations. Our discusions were limited to the end-user of the house screening. In this study, we did not interveiw or formally obtain the perceptions and experiences of the community leaders, policy makers such as the Zambia’s Ministry of Health and the facilitators of the house screening, namely the CHWs, carpenters and masons [41]. Future studies should obtain the views of these key stakeholders. Further, we do not rule out any influence that could have been excerted on the participants by the presence of the team of investigators from the study [41, 53].

## Conclusion

This study demonstrated that in rural south-east Zambia, closing eaves and screening windows and doors was a widely accepted intervention. Participants perceived that house screening reduced human-vector contact, reduced the malaria burden and nuisance biting from other potentially disease carrying insects. This adds to the growing body of evidence that house screening can be an effective and accepted supplementary vector-control tool. However, screened doors are more likely to be damaged, mainly by children, domestic animals, rust, and termites and largely on the bottom half. Based on these findings, we recommend PVC fibre glass for the screening material and a hard material for the bottom half of the screened door to increase durability.

### Electronic supplementary material

Below is the link to the electronic supplementary material.

Supplementary Material 1

Supplementary Material 2

Supplementary Material 3

## Data Availability

The datasets used and/or analysed during the current study have been made available as supplementary material. Further information can be obtained from the corresponding author on reasonable request.
