# Supplementary material for: Community perceptions, acceptability, and the durability of house screening interventions against exposure to malaria vectors in Nyimba district, Zambia
Source: BMC Public Health. 2024 Jan 24;24:285. doi: 10.1186/s12889-024-17750-4 (PMC10809574; doi:10.1186/s12889-024-17750-4)
Supplement: Supplementary file 1 — Supplementary Material 1 [file 12889_2024_17750_MOESM1_ESM.pdf]

Purpose of questionnaire: To assess the physical condition of the window and house screens and understand the main causes for tear.

Location: Nyimba district, Zambia

Date of interview: …………………… . **Household ID:** …………………………… …..

Village: …………………………………. **Zone:** ……………………………………

1. When was the house screened? (Mark the appropriate box)

☐ December 2019 ☐ January 2020

2. What is the condition of the windows? (Observe and mark the appropriate box. See definitions below)

☐ Intact ☐ Torn and/or has some holes ☐ Removed entirely.

i. **Intact:** the wire gauze does not have any visible damage or holes or tear larger than 2cm in diameter.
ii. **Torn and/or has some holes:** if the wire gauze is detached from the wooden plank or has a hole/s larger than 2cm in diameter.
iii. **Removed entirely:** The wire gauze is removed. If entirely removed, interview householder to determine the reasons.

Reasons for removal and/or tearing………………………………………………………

3. **Condition of the doors**

☐ Removed entirely ☐ Torn or has some holes ☐ Intact

*Definitions as shown above in 2.

4. If torn, which part of the door screen is most torn? (Observe and mark the appropriate box)

- [ ] Top
- [ ] Middle
- [ ] Bottom
- [ ] Wire mesh of the whole door is removed.

If removed entirely, interview the householder to determine the reasons behind removal.

Cause/Reasons for tear or removal..

Name of collector: . ...

Signature: ....
