# Supplementary material for: Community perceptions, acceptability, and the durability of house screening interventions against exposure to malaria vectors in Nyimba district, Zambia
Source: BMC Public Health. 2024 Jan 24;24:285. doi: 10.1186/s12889-024-17750-4 (PMC10809574; doi:10.1186/s12889-024-17750-4)
Supplement: Supplementary file 2 — Supplementary Material 2 [file 12889_2024_17750_MOESM2_ESM.pdf]

ADDITIONAL FILE 2

AFRO II PROJECT, NYIMBA DISTRICT

FOCUS GROUP DISCUSSIONS INTERVIEW GUIDES

INSTRUCTIONS

COMPOSITION: The Focus Group Discussion (FGD) is to be administered to a group of up to 12 respondents drawn from selected villages. These are villages where the AFRO II project conducted surveys previously and where some households had their houses screened. The FGDs will consist of 50% of respondents whose houses were screened and the other 50% whose houses were not screened. As much as possible there should be equal gender representation and fair different age group distribution in each group.

During each interview, there will be an interviewer and a note-taker. Interviews will also be recorded for further transcription. Respondents should be informed that their identity will remain anonymous and that they are free to participate or NOT. Also inform the group that the interviews will be recorded. The interview should on average take **about an hour**.

THE PURPOSE: Before the interview, make sure to introduce the team and the purposes of the discussion namely to get in-depth understanding from households that consented to their houses being screened and eaves closed as a malaria preventing method. Thank participants for their availability for the interview. The discussion should also assess knowledge, attitudes and practices on malaria control using house screening. Be warm and allow a free flow of the conversations without interruption.

Date of Interviews: ……………………(*dd/mm/yy*):   **Catchment Area**: Mkopeka/Nyimba Urban)………………………

Village:………………….. ……………………..

Interviewer details: Full Name……………………………………..Designation (write in full)…………………...Tel/mobile………………

<u>LIST OF PARTICIPANTS</u>

	**Full Name**	**Age**	**Gender**	**Was the house screened? Y/N**	**When was the house screened?** *Month/Year*
1					
2					
3					
4					
5					
6					
7					
8					
9					
10					
11					
12					

<u>QUESTIONS</u>

MALARIA PREVALENCE

1. How is the <u>malaria situation</u> in the village this year? How does this situation compare with other years in general?

2. How does this year compare with other years in terms of mosquito numbers in the houses?

3. What would you say is the proportion of children in the village who have had (confirmed) malaria this year? (using objects such as stones guide, the participants to estimate proportions in percentages)

4. What do you think are the reasons for the increase or decrease in the confirmed malaria cases in children?

    a. Optional /probing question: What proportion of children in the village have had malaria symptoms such as fever this year? (using objects such as stones guide the participants to estimate proportions in percentages)

    b. What are the reasons for the increase or decrease in the malaria symptoms such as fever in children this year?

MALARIA CONTROL

5. What methods are people in this village using to prevent mosquitoes entering their houses and to prevent malaria? (List all that are mentioned)

6. How effective are these methods? Have you faced any challenges in using these methods?

    a. **Optional/probing question**: What do you think are the challenges in using these methods?

KNOWLEDGE, ATTITUDES AND PRACTICES TOWARDS HOUSE SCREENING

*****If house screening was mentioned as a method of malaria control in the previous section, allow for a natural flow of questions.

7. Who can tell me (more) about house screening? Have you heard about house screening? What about closing eaves?

8. Where did you hear about house screening? [determine source of information]

9. When did you hear about house screening? [determine time frame of the source of information.]

10. Do you think house screening would help us prevent malaria? If so, how?

11. For those whose houses are screened, do you see any benefits of closing the eaves and house screening? What are the advantages of using the screens?

12. Are there any disadvantages of closing the eaves and screening the windows and the doors like we did? What are these disadvantages, if any?

13. For those whose houses were screened, what has been your experience with the house screens?

14. On a scale of 1 to10, how would you rate the effectiveness of house screening in preventing mosquito entry in the houses? (Allow the respondents to agree as a group)

15. On a scale of 1 to10, how would you rate the effectiveness of house screening in preventing malaria in children? (Allow the respondents to agree as a group)

DURABILITY OF THE SCREENS

16. What is the current condition of the screens on your windows and doors? (*Record number of respondents who say good out of total respondents available).*

17. Where does the damage to the house screens occur mostly?

18. What are the causes of the damage to the screens?

SUSTAINABILITY & WILLINGNESS TO PAY

19. Do people repair or replace the screens? If not, why are people NOT repairing the screens?

20. Question for those whose houses were SCREENED. Are people willing to maintain the screens in the absence of support from Government of Republic of Zambia (GRZ)/Donors/the AFRO II project? [ask with tact]

21. Would you recommend house screening to someone whose house was not screened?

22. *Question for those whose houses were NOT screened*. Are you willing to adopt house screening as a method of malaria prevention in your house?
23. *Question for those whose houses were NOT screened.* Would you be willing to adopt house screening in the absence of GRZ/Donor/AFRO II support.

Thank the participant. **THE END**
