# Supplementary material for: Community perceptions, acceptability, and the durability of house screening interventions against exposure to malaria vectors in Nyimba district, Zambia
Source: BMC Public Health. 2024 Jan 24;24:285. doi: 10.1186/s12889-024-17750-4 (PMC10809574; doi:10.1186/s12889-024-17750-4)

Additional file 3

Suggested changes to screened doors to increase durability.

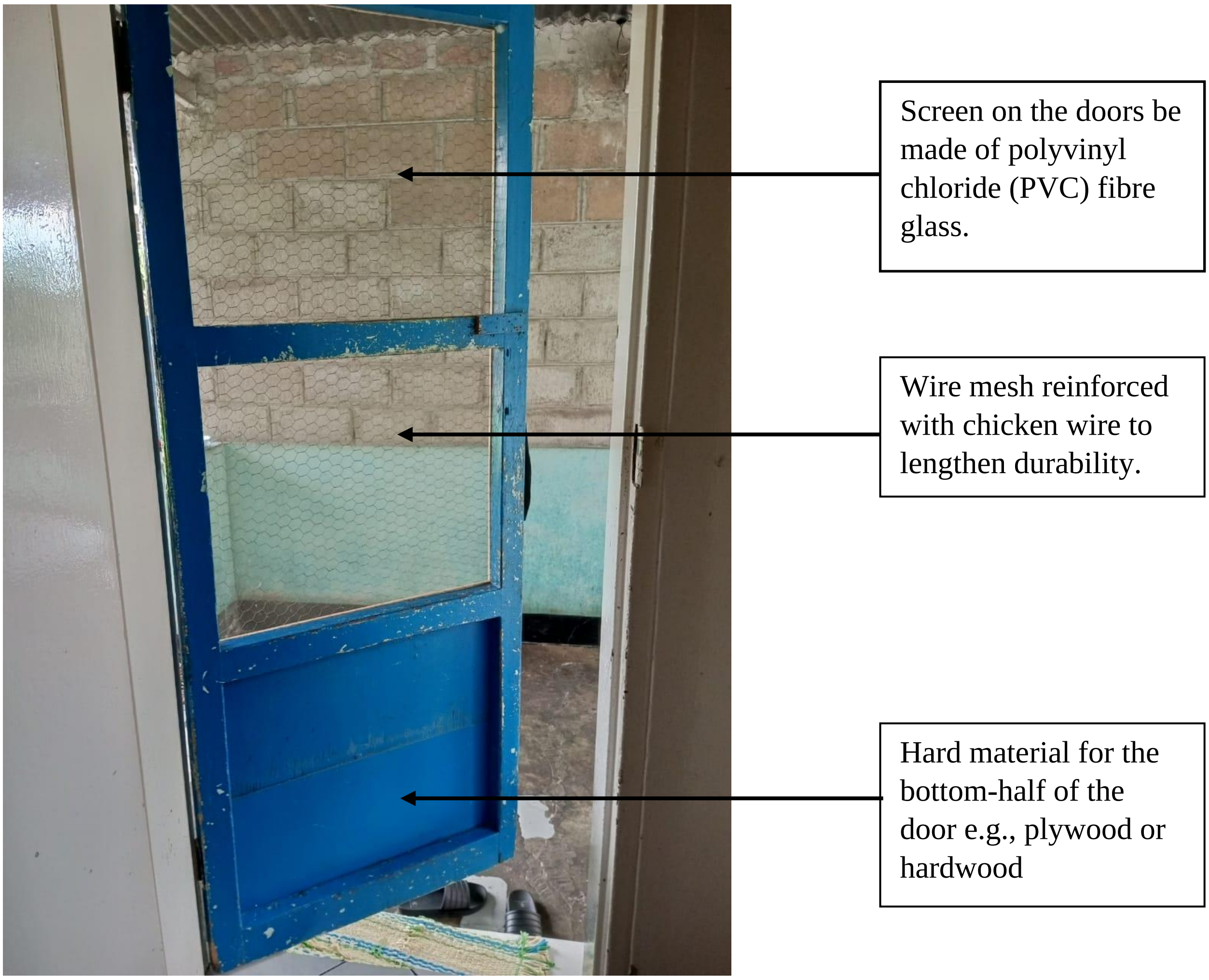

Supplement: Supplementary file 3 — Supplementary Material 3 [file 12889_2024_17750_MOESM3_ESM.pdf]
